# Food Safety Knowledge, Attitudes and Behavior among Dairy Plant Workers in Beijing, Northern China

**DOI:** 10.3390/ijerph15010063

**Published:** 2018-01-03

**Authors:** Yan Chen, Hua Ji, Li-Jun Chen, Rong Jiang, Yong-Ning Wu

**Affiliations:** 1 Key Laboratory of Food Safety Risk Assessment of Ministry of Health, China National Center for Food Safety Risk Assessment, Chaoyang District, Beijing 100021, China; 2 Food College, Shihezi University, Shihezi 832003, China; jihua229@126.com; 3 National Engineering Research Center for Maternal and Infant Dairy Health, Yinghai, Daxing District, Beijing 100163, China; jr2008@126.com

**Keywords:** attitudes, dairy plant worker, food safety, knowledge, behavior

## Abstract

The safety of milk and dairy products has always been one of the focuses of consumers, the food industry and regulatory agencies. The purpose of this study was to gain insight into the food safety knowledge, attitudes and behavior of dairy plant workers. A cross-sectional survey was performed between May and August 2015 in three dairy plants in Beijing, northern China. A total of 194 dairy plant workers were interviewed to collect information on food safety knowledge, attitudes and self-reported behavior. The 194 dairy plant workers interviewed showed a sufficient level of knowledge (mean score 34 on a scale from 0–58), perfect attitudes (mean score 17 on a scale from 0–18), and perfect behavior (mean score 38 on a scale from 8–40). Only 39% of workers correctly determined specific pathogens or diseases that could be conveyed through milk and dairy products. 24% of workers knew the correct method of washing hands. A significant positive association was observed between attitudes and knowledge (*p* < 0.001) as well as behavior (*p* < 0.01). Education level was positively and significantly associated with food safety knowledge, attitudes, and behavior (*p* < 0.05). Workers in dairy enterprises in northern China have relatively low levels of knowledge, yet satisfactory attitudes and behavior. The knowledge of microbial food hazards and hand hygiene remains an issue that needs to be emphasized in future training programs. Education level is a determinant of attitudes and behavior with regard to the proper handling of milk and dairy products.

## 1. Introduction

Milk and dairy products have increasingly become a major food in Chinese daily life; the Dietary Guidelines for Chinese recommend that most people include milk and dairy products in their daily diet [1]. The safety of milk and dairy products has always been one of the focuses of consumers, the food industry and regulatory agencies after the melamine incident in 2008. Although food adulteration is one of the concerns in China, the most important problem of food safety worldwide is foodborne diseases resulting from microorganisms [2], such as Campylobacteriosis, Listeriosis and Salmonellosis. Milk and dairy products are among the most important implicated food vehicles of foodborne diseases. A lot of pathogens can contaminate milk and dairy products and cause disease and death, such as *Brucella*, *Campylobacter*, *Listeria monocytogenes*, *Salmonella*, Shiga toxin-producing *Escherichia coli*, and *Shigella* [3]. Between 2003 and 2008, a total of 2795 foodborne disease outbreaks were reported to the China National Foodborne Diseases Surveillance Network, which resulted in 62,559 cases, 31,261 hospital admissions and 330 deaths in twelve provinces of China [4]. It is estimated that approximately 200 million cases of foodborne disease occurred in China annually [5].

China’s dairy industry chain, dominated by dairy processing enterprise, was divided into four modules, including feed, farm, dairy processing and consumer. Food production operators have the main responsibility to provide safe food to consumers and workers play a crucial role in the prevention of foodborne disease outbreaks. Therefore, knowledge and perception towards food safety among dairy plant workers are essential in ensuring the health of consumers. In that case, training programs are of particular importance for improving knowledge of dairy plant workers. In China food safety training among workers employed in the dairy industry in food safety is mandatory by food safety law: *A training system should be established and the food safety knowledge training should be conducted to all employees of the enterprise. In light of the different requirements of the positions, annual training plan should be developed and the corresponding training should be carried out. For special types of positions, workers should be certificated* [6].

Limited research on the food safety knowledge and behavior of dairy farm workers and plant workers has been published [7,8,9,10]. However, no research on the knowledge, attitudes and behavior among workers employed by dairy plants has been documented in China. Therefore, the objectives of this study were as follows: (1) to assess the knowledge, attitudes and behavior towards food safety among workers employed in dairy plants in northern China; and (2) to identify areas for improvements. This study provides baseline information to improve training programs for Chinese dairy plant workers.

## 2. Materials and Methods

### 2.1. Participants

A cross-sectional questionnaire survey was performed between May and August 2015 in three dairy processing plants in Beijing, northern China. Overall, about 2500 workers were employed in these plants. Beijing, as the capital city of China, most food is produced in and supplied from other provinces, and plants surveyed in this study representing major dairy processing plants in this area. The production level of these plants is to process 300 to 1000 tons of raw milk daily. On the front page of the questionnaire, a brief description of the purpose, the importance of the study, and the invitation for the worker to participate in the survey were included. With the help of the managers of the enterprises, a total of 194 workers directly involved in processing and packaging of milk and dairy products were conveniently sampled. Overall the response rate was 89%.

### 2.2. Data Collection

Questionnaires were distributed to all workers in the rest zone of the plants in order to be completed at the end of the rest. Upon completion of the signing of the informed consent form, workers were requested to finish a self-administrated questionnaire. The questionnaire was developed based on previous studies [10,11,12,13]. The questionnaire was piloted among 21 dairy plant workers and modified as needed and took about 10–15 min to complete.

The questionnaire had four sections including sociodemographic demographic information, knowledge, attitudes, and behavior with regard to food safety. In addition to sex, age, and education, the follow information was also collected: duration of employment in the food processing industry, enrollment in a food safety and foodborne disease training course, self-appraised food safety knowledge level and history of food poisoning.

Knowledge of food safety was evaluated using 29 questions, and the first 25 questions have three options (yes, no and unclear). A correctly answered question was assigned a score of 2 points otherwise a score of 0 was assigned. The scoring ranges from 0 to 58. Knowledge scores for individuals were counted and added up to obtain the total knowledge score. Participants who responded correctly to <50% of knowledge evaluation items were regarded as having poor knowledge of food safety.

For nine questions on attitude, participants have three options (agree, disagree and unsure). A correctly answered question was assigned a score of 2 points otherwise a score of 0 was assigned. The scoring ranges from 0 to 18. Attitude scores for individuals were counted and added up to obtain the total attitude score. Participants who responded correctly to <50% of attitude evaluation items were regarded as having poor attitude of food safety.

For eight questions on behavior, participants have five options (never, seldom, sometimes, usually and always). A five-point rating scale (1 to 5) was used for behavior questions with 5 representing the best behavior. The scoring ranges from 8 to 40. Behavior scores for individuals were counted and added up to obtain the total behavior score. Participants who scored <20 were regarded as having poor behavior of food safety.

### 2.3. Statistical Analysis

Data were entered using EpiData, version 3.1 (EpiData Association, Odense, Denmark) and analyzed using SPSS, version 16.0 (SPSS Inc., Chicago, IL, USA). Descriptive analysis including mean, standard deviation (SD) and frequency (%) was performed for all variables. Non-parametric comparisons (Mann-Whitney test, Kruskal Wallis test) were used to compare the mean sum of correct responses of knowledge, attitudes and behavior between gender and age groups. The associations between knowledge, attitudes and behavior scores and between the above-mentioned scores and demographic characteristics were tested using the Spearman rank correlation. Findings with a *p*-value less than 0.05 were regarded as statistically different.

### 2.4. Scientific Ethics

The study protocol was approved in 2015 by Human Ethics Committee at China National Center for Food Safety Risk Assessment (No. 2015007).

## 3. Results

### 3.1. Demographics

Demographic information of the respondents is shown in Table 1. A total of 194 dairy plant workers participated in the survey. The mean age of the respondents was 36 years of age (range 19–52 years of age; SD 8 years). Average duration of employment was seven years (SD 6 years). Ninety-six percent had attended food safety and foodborne disease training courses. Nearly 76% had received training for 3 times or more. Most of the respondents (87%) had very good/good knowledge of food safety. A history of food poisoning was reported by 13% of the respondents.

### 3.2. Knowledge

The mean general knowledge score was 34 (range 14–48 points; SD 6.9) with 78% above threshold level of 29 points (Table 2). There was no statistical difference in mean knowledge scores between age and gender groups (*p* > 0.05). The mean specific pathogen/disease knowledge score was 11 (range 0–20 points; SD 4.8) with 39% above threshold level of 12 points.

About 60% of the respondents knew that foodborne diseases may lead to miscarriage in pregnant women. Most of the respondents (60–90%) recognized that preschoolers, pregnant women and older people, and HIV-positive persons and cancer patients are at high risk of becoming ill due to the pathogenic microorganisms in food. A low proportion of the respondents knew that people who frequently eat outside (7%) and teenagers (28%) are not the population at high risk.

In total, 52% and 62% of the respondents correctly answered that anthrax and brucellosis could be conveyed through milk and dairy products, respectively. However, the corresponding figure was only 4% for bovine viral diarrhea and 6% for Johne’s disease, both of these diseases could not be conveyed through milk and dairy products. The percentages of correct responses of *E. coli* O157:H7, *Salmonella* spp., *Staphylococcus aureas*, *L. monocytogens*, *Campylobacter* spp. and noroviruses were 88%, 74%, 73%, 56%, 53% and 48%, respectively. However, correct responses to *Clostridium botulinum* (8%) and *Toxoplasma gondii* (7%) were generally low.

Nearly 80% of respondents knew the optimal method to clean fresh vegetables and fruits (with cool running water). However, only 24% knew the best way to wash hands, which includes wetting, applying soap, rubbing for 20 s, rinsing and drying hands. Approximately 60% of the respondents could not specify the recommended freezer temperature (−18 °C). However, 68% of the respondents knew the maximum refrigeration temperature (4 °C) to maintain the safety of foods.

### 3.3. Attitudes

The mean attitude score was 17 (SD = 1.8) with 99% above 9 (Table 3). There was no statistical difference in mean attitude scores between age and gender groups (*p* > 0.05). More than 96% of the respondents agreed that use of work uniform, masks and caps, raw foods stored separately from cooked food, food safety training for workers and monitoring of refrigerator temperatures have a great influence on decreasing risk of food contamination. 8% of the respondents disagreed with the statement that workers with wounds on their hands should not handle food with their bare hands.

### 3.4. Behavior

All respondents had a total self-reported behavior score above 20. The mean behavior score was 38 (SD = 3.2), ranging from 24 to 40 points (Table 4). There was no statistical difference in mean behavior scores between age and gender groups (*p* > 0.05).

More than 85% of the respondents always wore work uniform and cap, never wore watch and decoration, always washed hands after handling waste/garbage or after using the toilet, never smoke or ate/drank during their work except for breaks. However, among the 193 dairy plant workers, only 64% (123/193) of them always wore masks, whereas 7% (14/193) seldom or never wore masks while working.

### 3.5. Correlation Results

Significant weak associations were observed between attitudes with knowledge (rho = 0.261, *p* < 0.001) and behavior (rho = 0.200, *p* < 0.01). There was no significant association between knowledge and behavior. 

Education level was significantly positively related with knowledge (rho = 0.221, *p* < 0.05), attitudes (rho = 0.159, *p* < 0.05), and behavior (rho = 0.173, *p* < 0.05), though the associations were weak. There was no significant association between duration of employment with knowledge, attitudes, and behavior.

## 4. Discussion

This is the first study to assess knowledge, attitudes and behavior toward food safety among dairy plant workers in China. Our findings demonstrated that workers employed in the three dairy enterprises in northern China have relatively lower-level of knowledge, yet satisfactory attitudes and behavior.

The majority (78%) of the dairy plant workers answered correctly to more than 50% of the questions in the knowledge section. However, their knowledge of the specific diseases or pathogens that could be conveyed through milk and dairy products, was relatively low. Previous studies had showed that dairy farm workers did not have enough knowledge of foodborne diseases or pathogens [10].

About 50% of the respondents in this study were unaware that norovirus could spread from milk and dairy product to humans, while *C. botulinum* and *T. gondii* could not. More than 40% of workers were unsure whether anthrax, *Campylobacter* or *L. monocytogens* could convey through milk and dairy products to humans. This is surprising as the majority of dairy plant workers participated in food safety training courses. There is a need to develop more tailored training programs for dairy plant workers. Future research should assess the effectiveness of mandatory continued education activities in food safety for dairy plant workers.

*E. coli* O157:H7 is one of the most virulent pathogens responsible for foodborne disease globally. Cattle are the main host for *E. coli* O157:H7 and outbreaks are frequently attributed to the ingestion of contaminated milk and dairy products of cattle origin [14]. In 1999, a large-scale *E. coli* O157:H7 outbreak in China had resulted in 195 hospital admissions due to hemolytic uremic syndrome and 177 deaths [15]. In the present study, the proportion of respondents who identified *E. coli* O157:H7 as a milk-borne pathogen was greater than those who identified other pathogens as milk-borne. This difference could be due to the fact that the issue of *E. coli* O157:H7 has been highly publicized through media in China.

About 40% of the respondents in this study were unaware that foodborne diseases might cause miscarriage in pregnant women. Meanwhile, 44% of the respondents were unaware that *L. monocytogenes* could spread from milk and dairy products to humans. Although affected pregnant women usually have mild symptoms, *L. monocytogenes* can lead to severe outcomes for fetus or newborn infant; infection in utero can cause preterm labor or spontaneous miscarriage and stillbirth [16]. Southwestern Ontario artisan cheese makers perceived *L. monocytogenes* to be the most harmful biological hazard to the health of consumers and to their businesses [8].

The proportions of correct answers to optimal refrigeration temperature for food storage (68%) and freezing of food (38%) varied greatly in our study. The figures were lower than found in other studies in Lebanon (78% and 55%) [17] and in Romania (76%, respectively) [18]. It is worth noting that there may not be a single “maximum refrigeration temperature in order to maintain the safety of food” (Table 2, Question 28). As *L. monocytogenes* can grow in permissive foods even when these foods are stored at 4 °C. Correspondingly, the “monitoring of refrigeration temperatures” may have no effect on “decreasing the risk of food contamination” (Table 3, Question 7).

In several studies on foodborne outbreaks, the hands of workers have been determined as the origin of pathogens in the involved food vehicles. Hand washing is the easiest and most efficient manner to remove pathogens on the hands [19]. In line with findings from previous studies in Lebanon and Romania [17,18], almost all respondents (nearly 90%) in our study always wash hands after handling waste/garbage, or after toilet visits. However, only 24% of respondents in the present study knew the most hygienic hand-washing steps, lower than previous studies [18,20].

The study highlights the correlation between the level of education and the level of food safety knowledge, attitudes and behavior. Previous studies showed that workers with higher educational levels have a significantly higher food safety knowledge [13].

Compared with the perfect attitudes and behavior, the relatively lower-level knowledge presented in the study suggests the inefficiency of the food safety training programs, indicating the Beijing dairy enterprises need to further improve training on employee food safety knowledge. An efficient training program on food safety knowledge should at least include materials on major foodborne pathogens, high-risk population, safe storing temperature, and appropriate cleaning and hygiene procedures. Without well-trained workers who are aware of the importance of safety procedures in the food processing, it is difficult to implement and maintain a functional food safety system.

The limitations of this study are similar to other research on knowledge, attitudes and behaviors. The inherent limitation of such research is that the response may be influenced by social expectations. The sample size regarding the number of participants is sufficient, but they were recruited from only three plants. As the study is not anonymous, there may be an influence between the involvements of company management upon the collection of the questionnaires. Another serious limitation is that the sampling of participants was “convenience sampling”. While the survey may not be representative of all dairy plant workers, it does indicate problems of professional knowledge and behaviors in a high percentage of those surveyed and the work does suggest problems that need to be addressed.

The focus of this study is on dairy plant workers in Beijing, China, so the findings should not be extended to all dairy plant workers in the country. Besides, dairy farming is also an important part of the dairy industry, therefore, similar assessment of food safety knowledge, attitudes and behavior is required for dairy farm workers in the future.

## 5. Conclusions

We conclude that workers employed in dairy enterprises in northern China have a relatively lower level of knowledge, yet satisfactory attitudes and behavior. The knowledge of microbial food hazards and hand hygiene remains an issue that needs to be emphasized in food safety training programs in the Chinese dairy enterprises. Education level is a determinant of attitudes and behavior with regard to the proper handling of milk and dairy products.

## Figures and Tables

**Table 1:** Demographic characteristics of the respondents.

Variable	N ^a^	%
Sex	193	
Male	83	43
Female	110	57
Age (years)	190	
≤20	5	3
21–30	48	25
31–40	74	39
41–50	60	32
≥51	3	2
Education	194	
Primary and secondary school	72	37
High school and above	122	63
Years working in food processing industry	192	
1–5	114	59
6–10	54	28
>10	24	13

^a^ Number of respondents.

**Table 2:** Food safety related knowledge among dairy plant workers.

Statements		N ^a^	Correct Answers (%)
1	Hand washing before work decreases the likelihood of food being contaminated	194	100
2	Handling and cleaning of the equipment properly decreases the likelihood of food being contaminated	194	100
3	Consuming food or beverages inside the working space increases the likelihood of food being contaminated	194	94
4	Foodborne illnesses may lead to miscarriage in pregnant women	193	61
5	In the time of infectious eye illness, it is necessary to stay off the job	192	91
6	In the time of infectious skin illness, it is necessary to stay off the job	194	99
7	Compared to most people, preschool children have a higher possibility to become ill due to the pathogenic microorganisms in food	192	93
8	Compared to most people, teenagers have a higher possibility to become ill due to the pathogenic microorganisms in food	193	28
9	Compared to most people, pregnant women have a higher possibility to become ill due to the pathogenic microorganisms in food	192	84
10	Compared to most people, older people (age 60 and over) have a higher possibility to become ill due to the pathogenic microorganisms in food	192	89
11	Compared to most people, people who are HIV positive have a higher possibility to become ill due to the pathogenic microorganisms in food	192	63
12	Compared to most people, cancer patients have a higher possibility to become ill due to the pathogenic microorganisms in food	193	63
13	Compared to most people, people who frequently eat at restaurants or get take-out food have a higher possibility to become ill due to the pathogenic microorganisms in food	193	7
14	Can anthrax be spread from milk and dairy products to humans?	193	52
15	Can *Clostridium botulinum* be spread from milk and dairy products to humans?	192	8
16	Can bovine viral diarrhea be spread from milk and dairy products to humans?	192	4
17	Can Brucellosis be spread from milk and dairy products to humans?	192	62
18	Can *Campylobacter* spp. be spread from milk and dairy products to humans?	191	53
19	Can *Escherichia coli* O157:H7 be spread from milk and dairy products to humans?	193	88
20	Can Johne’s disease be spread from milk and dairy products to humans?	189	6
21	Can *Listeria monocytogens* be spread from milk and dairy products to humans?	193	56
22	Can noroviruses be spread from milk and dairy products to humans?	192	48
23	Can *Salmonella* spp. be spread from milk and dairy products to humans?	194	74
24	Can *Staphylococcus aureas* be spread from milk and dairy products to humans?	193	73
25	Can *Toxoplasma gondii* be spread from milk and dairy products to humans?	189	7
26	Which of the following methods of cleaning fresh vegetables and fruits is the best to prevent food poisoning?	193	78
27	In order to guard against food poisoning, what is the recommended temperature freezer should be?	193	38
28	What is the maximum refrigeration temperature in order to maintain the safety of food?	192	68
29	Which of the following is the most efficient hand wash method?	187	24
% Passing ^b^		78%	
Overall score (mean ± SD) ^c^		34 ± 6.9	

SD, Standard deviation. ^a^ Number of respondents. ^b^ The percentage of worker who answered correctly more than 50% of the questions. ^c^ The score varied between 0 and 58.

**Table 3:** Food safety related attitudes among dairy plant workers.

Statements		N ^a^	Correct Answers (%)
1	Wearing work uniform is crucial in decreasing the likelihood of food being contaminated	194	97
2	Wearing masks is crucial in decreasing the likelihood of food being contaminated	194	99
3	Wearing caps is crucial in decreasing the likelihood of food being contaminated	194	99
4	Workers with wounds on their hands should not handle foods with their bare hands	193	92
5	Raw foods should be stored separately from cooked foods to decrease risk of food contamination	193	100
6	Food safety training for workers is a major problem in decreasing risk of food contamination	194	97
7	It is essential to measure refrigerator temperature at regular intervals to decrease the likelihood of food being contaminated	194	97
8	Health condition of the workers should be assessed before employment	194	95
9	Foodborne illnesses can impose adverse healthy and economic impacts on the society	194	94
% Passing ^b^		99%	
Overall score (mean ± SD) ^c^		17 ± 1.8	

SD, Standard deviation. ^a^ Number of respondents. ^b^ The percentage of worker who answered correctly more than 50% of the questions. ^c^ The score varied between 0 and 18.

**Table 4:** Food safety related behavior among dairy plant workers.

Statements		N ^a^	Answers, %				
			Never	Seldom	Sometimes	Usually	Always
1	Do you wear work uniform while working?	193	0	0	1	9	90
2	Do you wear a mask while working?	193	3	4	14	15	64
3	Do you wear a cap while working?	194	2	1	2	10	86
4	Do you wear watch and decoration while working?	194	94	1	0	2	4
5	Do you wash your hands after handling waste/garbage?	194	1	0	1	10	88
6	Do you wash your hands after using the toilet when you go back to work?	192	2	0	1	10	88
7	Do you consume food or beverages inside the working space?	194	90	4	3	2	1
8	Do you smoke inside the working space?	193	96	2	1	1	1
% Passing ^b^		100%					
Overall score (mean ± SD) ^c^		38 ± 3.2					

SD, Standard deviation. ^a^ Number of respondents. ^b^ The percentage of worker whose score was equal and above 20. ^c^ The score varied between 8 and 40.
